# Oxaloacetate and Ketone Bodies Synergistically Promote Myoblast Differentiation in L6 Cells

**DOI:** 10.3390/molecules30102101

**Published:** 2025-05-09

**Authors:** Yuji Onuki, Naoki Nanashima, Yutaro Sasaki, Akiko Kojima-Yuasa, Toshio Norikura

**Affiliations:** 1 Graduate School of Health Sciences, Aomori University of Health and Welfare, Aomori 030-8505, Japan; 2 Graduate School of Human Life and Ecology, Osaka Metropolitan University, Osaka 558-8585, Japan; kojima-yuasa@omu.ac.jp

**Keywords:** myoblast, myotube, differentiation, myogenesis, ketone bodies, oxaloacetate

## Abstract

Malnutrition and aging are major factors that inhibit myoblast differentiation, leading to a decline in muscle function and contributing to sarcopenia development. This study aimed to elucidate the role of nutrients in myoblast differentiation by establishing a culture system at physiological glucose levels and investigating the effects of ketone bodies and oxaloacetate. We successfully cultured myoblasts at physiological glucose concentrations in a hydrophobic membrane filter-equipped culture flask. Under these conditions, ketone bodies and oxaloacetate synergistically upregulated myogenic differentiation markers (*Lmod2* and *Ckm*), indicating enhanced differentiation. Additionally, oxaloacetate upregulated mitochondrial biogenesis markers (mitochondrial DNA copy number and Cs), whereas ketone bodies promoted Akt phosphorylation, a key regulator of differentiation, via the PI3K/Akt/mTOR pathway. These results suggest that the intake of ketone bodies and oxaloacetate effectively prevents sarcopenia by synergistically promoting myoblast differentiation via distinct molecular mechanisms, suggesting a potential new nutritional strategy.

## 1. Introduction

Skeletal muscles are indispensable for voluntary movement and have been reported to be involved in various functions, such as metabolism and endocrine regulation. Skeletal muscle maintains its mass through a continuous cycle of damage and regeneration, and any disruption in this balance results in the loss of muscle mass and the development of various diseases. In recent years, sarcopenia, which is the loss of muscle mass due to aging and malnutrition, has become a significant societal issue as it contributes to the increase in the number of elderly individuals requiring care in an aging society.

Normal skeletal muscle growth and repair involve the activation of quiescent satellite cells into myoblasts, which proliferate and differentiate into multinucleated myotubes [1]. Although the association of sarcopenia with a diminished differentiation potential of myoblasts has been established [2], the precise underlying mechanisms remain unclear.

The medium in cell culture, similar to the interstitial fluid in vivo, provides nutrients to cells in vitro. The concentration of nutrients is considerably higher in high-glucose Dulbecco’s modified eagle medium (DMEM), which is frequently used for culturing myoblasts, than in the interstitial fluid [3]. This adjustment compensates for the rapid nutrient consumption by cells in vitro, enabling sustained cell culture without requiring frequent medium changes [4]. However, high-glucose DMEM can alter the metabolism of cultured cells, inducing metabolic phenotypes that may not accurately reflect in vivo conditions [5]. This deviation presents limitations in the correct assessment of the functions of the nutrients added to the medium.

Ketone bodies are produced from acetyl-CoA, which is generated through the β-oxidation of fatty acids, primarily under carbohydrate-deficient conditions, and serve as an alternative energy source to glucose [6]. When oxaloacetate is diverted for gluconeogenesis, excess acetyl-CoA is converted into ketone bodies due to reduced entry into the citric acid cycle. Ketone bodies are classified into three main types—acetoacetate, β-hydroxybutyrate, and acetone—with only acetoacetate and β-hydroxybutyrate being metabolically active. Blood ketone levels can reach 1–3 mM during a ketogenic diet [7], approximately 7 mM during prolonged starvation [8], and up to 25 mM in diabetic ketoacidosis [9].

In the 1920s, the ketogenic diet was developed as a treatment for refractory epilepsy, with fat intake comprising approximately 90% of the total calories [10]. Additionally, it has become a common dietary approach to achieve weight loss [11]. However, a ketogenic diet is extremely unbalanced in terms of nutritional composition and is significantly different from a typical diet. The development of accessible ketone supplements has enabled the achievement of nutritional ketosis without extreme dietary restrictions [12]. According to previous studies, the acute administration of β-hydroxybutyrate to rats improved their athletic performance [13]. Furthermore, intervention studies in athletes have reported that consuming drinks containing ketone bodies improved their endurance performance [14]. However, some reports have indicated the negative effects of exogenous ketone supplements on athletic performance [15].

Nutritional ketosis induced by ketone supplements and a ketogenic diet is considered a physiological state with lower blood ketone concentrations than diabetic ketoacidosis without pH changes [16]. The precise mechanism by which nutritional ketosis influences athletic performance remains unclear, although the potential involvement of citric acid cycle activation has been suggested [13]. Thus, we hypothesized that if ketone bodies are metabolized into acetyl-CoA and the citric acid cycle is activated, then combining metabolites related to the citric acid cycle may further improve athletic performance.

Previous myoblast culture experiments have been conducted under high glucose concentrations to simulate a diabetic ketoacidosis-like state. The metabolic state of the cells would also change if the nutrient concentrations in the culture medium differed from physiological levels. Therefore, this study aimed to establish culture conditions for myoblasts that mimic physiological nutritional conditions and to determine the effects of ketone bodies and oxaloacetate on myoblast differentiation.

## 2. Results

### 2.1. Changes in Glucose Concentration in Culture Medium over Time

**Figure 1:**
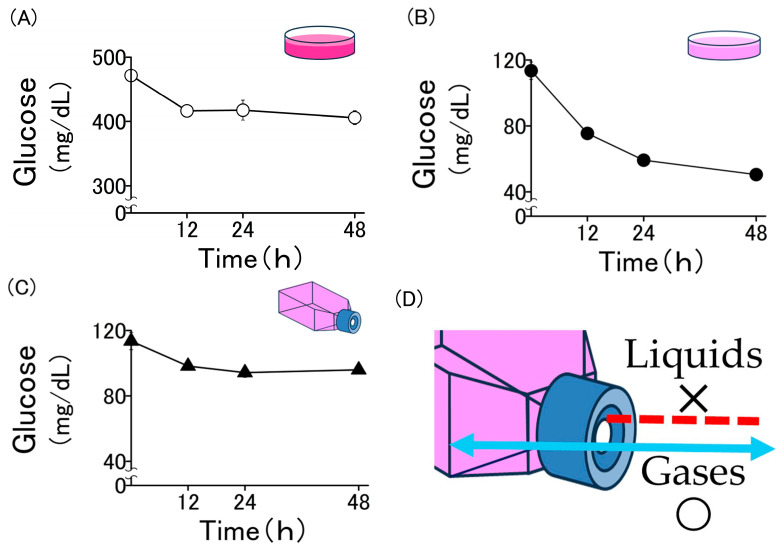
Glucose concentration in the medium over the incubation period. (**A**) The cells were incubated with high-glucose DMEM in dishes. (**B**) The cells were incubated with low-glucose DMEM in dishes. (**C**) The cells were incubated with low-glucose DMEM in flasks. (**D**) The culture flask equipped with the hydrophobic membrane filter used in this experiment allows only gases to pass through. Values are expressed as the mean ± SD of three independent experiments.

DMEM, one of the most commonly used culture media for experiments involving cultured cells, is commercially available in high- (450 mg/dL) and low-glucose (100 mg/dL) versions. To establish culture conditions with physiological glucose concentrations, we examined the change in glucose concentration (glucose consumption) over time when myoblasts were cultured in these media. In dish cultures with a high-glucose medium (450 mg/dL), the glucose concentration remained above 400 mg/dL even after 48 h of incubation. This corresponds to a state of extreme hyperglycemia in humans and significantly deviates from physiological culture conditions (Figure 1A). In dish cultures with low-glucose medium (100 mg/dL), the glucose concentration remained within the physiological range (80–110 mg/dL) for the first 12 h but dropped below 60 mg/dL after 48 h of incubation, indicating severe hypoglycemia (Figure 1B). In flask cultures with a low-glucose medium (100 mg/dL), the decline in glucose concentration over time was more gradual. Even after 48 h of incubation, the glucose concentration remained at approximately 100 mg/dL, thus maintaining physiological levels (Figure 1C). Thus, although glucose concentrations varied over time depending on the culture conditions, these changes were not due to intentional adjustments but rather resulted from cellular consumption.

### 2.2. L6 Cells Differentiation Is Enhanced by Ketone Bodies in a Concentration-Dependent Manner

**Figure 2:**
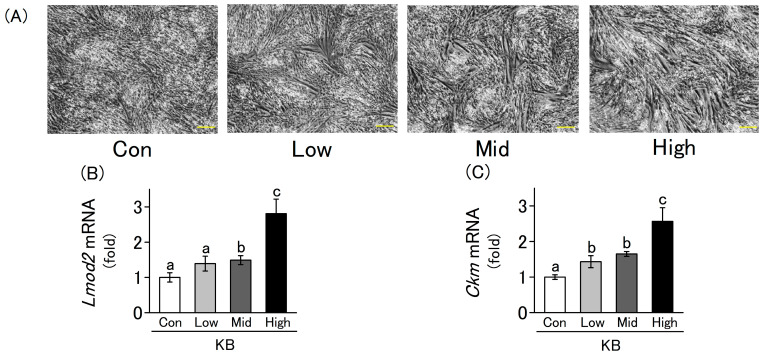
Effect of ketone bodies (KB) on the differentiation of L6 cells. The cells were incubated with differentiation medium for 4 d with or without KB. Con (Control), Low (0.5 mM LA and 2.0 mM HB), Mid (1.0 mM LA and 4.0 mM HB), and High (2.0 mM LA and 8.0 mM HB) indicate different KB concentrations. (**A**) The differentiation state of L6 cells was evaluated using an optical microscope at a magnification of 40× (scale bar = 200 µm). (**B**,**C**) Relative mRNA expression levels of differentiation markers (*Lmod2* and *Ckm*) were quantified using RT-qPCR. Values are expressed as the mean ± SD of three independent experiments. Data were analyzed using one-way analysis of variance (ANOVA) followed by Tukey–Kramer multiple comparison test. Different letters above the bars indicate statistically significant differences at the 5% level.

During myogenesis, myoblasts proliferate, differentiate, and fuse to form multinucleated myotubes, which further develop into myofibers. During this period, the expression levels of the myogenic differentiation marker genes increase. Therefore, we evaluated the differentiation state of myoblasts through microscopic observation and gene expression analysis using RT-qPCR. L6 cells were cultured in a differentiation medium for 4 d in the presence or absence of ketone bodies. Cell differentiation was promoted in a concentration-dependent manner, with higher concentrations of ketone bodies leading to more pronounced morphological changes such as elongated myotube formation (Figure 2A). The mRNA expression levels of the differentiation markers *Lmod2* and *Ckm* were quantified using RT-qPCR. Their expression levels increased significantly in a concentration-dependent manner upon the addition of ketone bodies to the culture medium (Figure 2B,C). A ketogenic diet typically elevates circulating β-hydroxybutyrate levels to approximately 0.5–3.0 mM, resulting in a β-hydroxybutyrate-to-acetoacetate ratio of about 4:1 [9]. Therefore, the results of this study suggest that ketone bodies promote myoblast differentiation under culture conditions that mimic the physiological blood concentrations observed during a ketogenic diet. These results suggest that ketone bodies enhance L6 cell differentiation at both the morphological and gene expression levels (*p* < 0.05).

### 2.3. Fluctuations in the Expression of Myogenic Marker Genes Due to the Addition of Ketone Bodies and Metabolites Related to the Citric Acid Cycle

**Figure 3:**
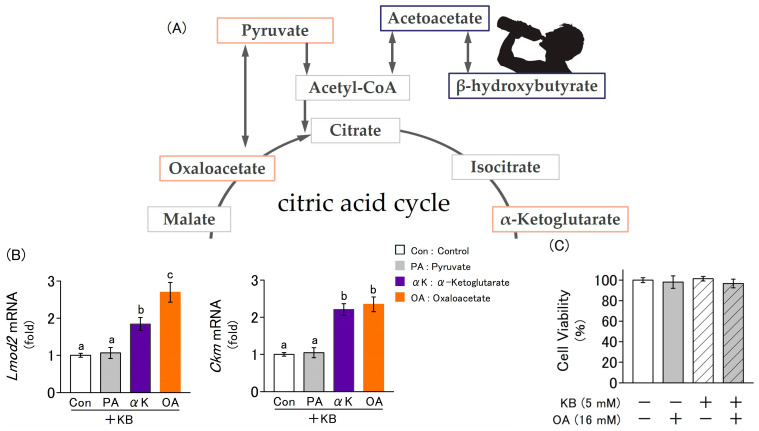
Interactive effects of KB and citric acid cycle-related metabolites on L6 cells differentiation. Cells were cultured for 4 d in differentiation medium supplemented with or without KB (1 mM LA and 4 mM HB), PA (16 mM pyruvate), αK (16 mM α-ketoglutarate), and OA (16 mM oxaloacetate). (**A**) Metabolic pathways of KB (acetoacetate and β-hydroxybutyrate) and intermediate metabolites of the citric acid cycle. (**B**) Relative mRNA expression levels of differentiation markers (*Lmod2* and *Ckm*) were quantified using RT-qPCR. (**C**) Cell viability was determined using neutral red assay. Values are expressed as the mean ± SD of three independent experiments. Data were analyzed using one-way ANOVA followed by Tukey–Kramer multiple comparison test. Different letters above the bars indicate statistically significant differences at the 5% level.

L6 cells were cultured in a differentiation medium for 4 d, supplemented with ketone bodies combined with one of the following citric acid cycle-related metabolites: pyruvate (PA), α-ketoglutarate (αK), or oxaloacetate. Among these metabolites, αK and oxaloacetate significantly increased the expression levels of *Lmod2* and *Ckm*, as quantified by RT-qPCR (Figure 3B). This result indicates that together with ketone bodies, αK and oxaloacetate further promote myoblast differentiation. To evaluate the cytotoxicity of ketone bodies and oxaloacetate in the culture medium, a Neutral Red assay was used to assess cell viability after 24 h of culture in a medium supplemented with ketone bodies (5 mM) and oxaloacetate (16 mM). No significant changes were observed in any group (Figure 3C).

### 2.4. Synergistic Effect of Ketone Bodies and Oxaloacetate on the Differentiation of L6 Cells

**Figure 4:**
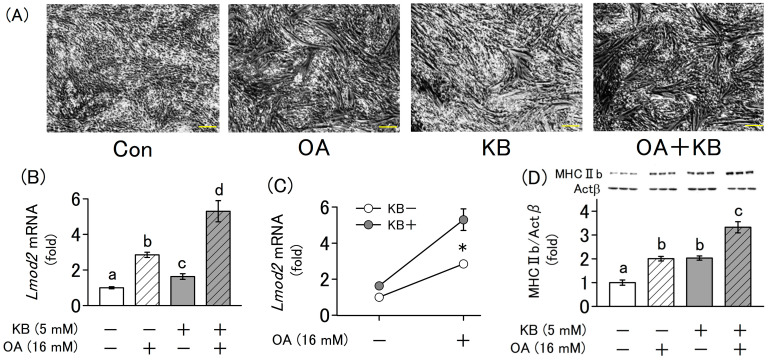

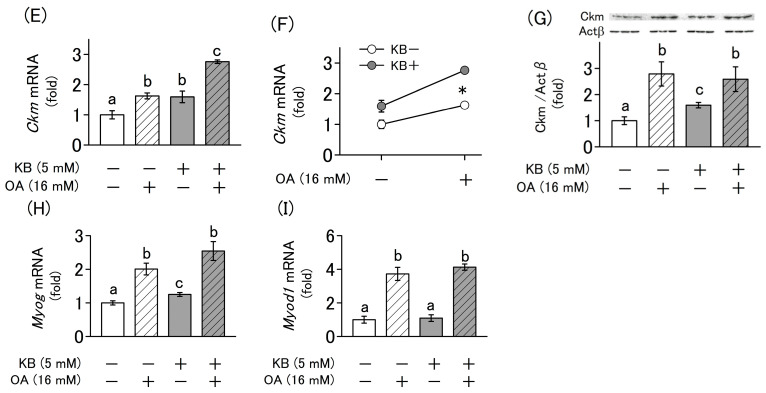
Interactive effect of KB and OA on the differentiation of L6 cells. Cells were cultured for 4 d in differentiation medium supplemented with or without KB (1 mM LA and 4 mM HB) and OA (16 mM). (**A**) The differentiation state of L6 cells was evaluated using an optical microscope at a magnification of 40× (scale bar = 200 µm). (**B**,**E**,**H**,**I**) Relative mRNA expression levels of differentiation markers (*Lmod2*, *Ckm*, *Myog* and *Myod1*) were quantified using RT-qPCR. (**C**,**F**) The interactive effect of ketone bodies (KB) and oxaloacetate (OA) on differentiation was evaluated using two-way ANOVA. An asterisk (*) indicates a significant interaction between KB and OA (*p* < 0.05). (**D**,**G**) The protein levels of MHC IIb and Ckm were measured using Western blotting, with Actb as the internal control. Values are expressed as the mean ± SD of three independent experiments. Data were analyzed using one-way ANOVA followed by Tukey–Kramer multiple comparison test. Different letters above the bars indicate statistically significant differences at the 5% level.

The simultaneous administration of ketone bodies and oxaloacetate significantly enhanced muscle differentiation compared with the administration of each compound alone, as shown by morphological changes (Figure 4A) and mRNA expression levels of *Lmod2, Ckm* (Figure 4B,E). Additionally, the increase in the expression of these genes by the simultaneous addition of ketone bodies and oxaloacetate was found to have a synergistic effect (*p* < 0.05), as revealed by two-way ANOVA (Figure 4C,F). Furthermore, gene expression levels of the traditional differentiation marker genes Myog and Myod1 were also significantly increased (Figure 4H,I).

In this experiment, the protein expression levels of MHC IIb and Ckm, which are markers of myotube formation, were measured using Western blotting. Similar to the mRNA expression levels, the simultaneous addition of oxaloacetate and ketone bodies significantly increased the levels of these proteins (Figure 4D,G).

### 2.5. Effects of Ketone Bodie and Oxaloacetate on the Regulation of Mitochondrial Biogenesis and Cellular Homeostasis in L6 Cells

**Figure 5:**
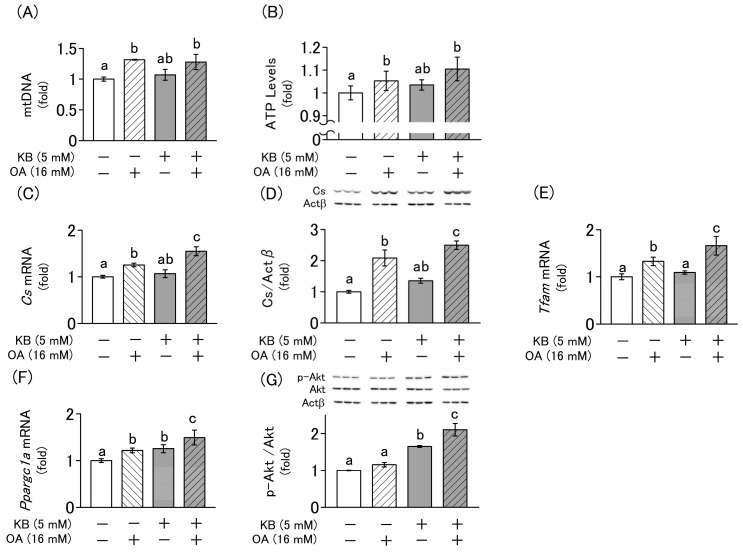
Interactive effects of KB and OA on the regulation of mitochondrial biogenesis and cellular homeostasis in L6 cells. Cells were cultured for 4 d in differentiation medium supplemented with or without KB (1 mM LA and 4 mM HB) and OA (16 mM). (**A**) Relative mtDNA copy number, defined as the ratio of mitochondrial DNA-encoded *Mt-CO1* to nDNA-encoded *Actb*, was determined using qPCR. (**B**) ATP contents in L6 cells were assessed using the CellTiter-Glo^®^ 2.0 assay. (**C**,**E**,**F**) Relative mRNA expression levels of a mitochondrial biogenesis marker gene (*Cs, Tfam and Ppargc1a*) was quantified using RT-qPCR. (**D**,**G**) The protein levels of Cs and p-Akt were measured using Western blotting, with Actb as the internal control. The levels of p-Akt were normalized to that of Akt. Values are expressed as the mean ± SD of three or six independent experiments. Data were analyzed using one-way ANOVA followed by Tukey–Kramer multiple comparison test. Different letters above the bars indicate statistically significant differences at the 5% level.

Because mitochondrial biogenesis decreases with age, it has been implicated in reduced energy metabolism and muscle differentiation. Thus, we investigated the influence of ketone bodies and oxaloacetate on mitochondrial biogenesis, intracellular ATP content, and protein kinase B (Akt) phosphorylation to elucidate the mechanisms of myoblast differentiation. The addition of oxaloacetate significantly increased the mitochondrial DNA (mtDNA) copy number (Figure 5A), ATP content (Figure 5B), and the mRNA and protein expression levels of *Cs*, *Tfam*, and *Ppargc1a*, which are indicators of mitochondrial biogenesis. However, no significant changes were observed with the addition of ketone bodies (Figure 5C–F). In contrast, the phosphorylation level of Akt, which is involved in myocyte differentiation via the PI3K/Akt/mammalian target of rapamycin (mTOR) pathway, significantly increased upon the addition of ketone bodies. However, no significant change was observed with the addition of oxaloacetate (Figure 5G).

## 3. Discussion

The concentration of ketone bodies in the blood increases primarily during starvation, low-carbohydrate diet intake, and severe diabetes [17]. The increase in blood ketone levels that occurs in diabetes (diabetic ketoacidosis) is generally not considered a favorable metabolic state because it is accompanied by a decrease in blood pH and an increase in blood glucose levels. In contrast, nutritional ketosis, which has gained attention in recent years, is a metabolic state in which blood ketone levels reach 0.5–5 mM while maintaining a normal glucose concentration and pH due to a low-carbohydrate diet or the intake of ketone supplements [18]. A recent study reported that ketone supplementation elevates blood ketone levels and may contribute to enhanced athletic performance [14]. However, the underlying mechanism remains unclear.

Because glucose is utilized as an energy source for cell proliferation, culture media with high glucose concentrations are typically used for cell growth [19,20]. However, DMEM containing a high glucose concentration (342 mg/dL) has been reported to inhibit the proliferation of satellite cells, which are muscle-specific stem cells [21]. Therefore, to elucidate the physiological effects of nutritional ketosis on muscles, culture conditions should be established for myoblasts at physiological glucose concentrations, as conventional culture conditions for myoblasts (glucose 450 mg/dL) serve as models for diabetic ketoacidosis.

In this study, we established a culture model with a physiological glucose concentration using a flask equipped with a recently developed hydrophobic membrane filter and by increasing the amount of culture medium per cell 10-fold compared with that in conventional methods (Figure 1D). Under these conditions, the consumption of glucose by the cells remained unchanged, and fluctuations in its concentration in the culture medium were minimal. Using this model, we demonstrate for the first time that the addition of ketone bodies under physiological glucose conditions promotes myoblast differentiation.

Furthermore, the intake of ketone supplements has been previously reported to im-prove exercise performance via the activation of the citric acid cycle [13]. In the first step of the citric acid cycle, oxaloacetate reacts with acetyl-CoA to produce citrate (Figure 3A). Oxaloacetate is a key intermediate in the citric acid cycle, which facilitates energy metabolism and functions as a gluconeogenic precursor, contributing to the maintenance of blood glucose homeostasis under carbohydrate-restricted conditions. Ketosis is a metabolic state characterized by reduced carbohydrate utilization and increased lipid metabolism. Under these conditions, the excess acetyl-CoA generated from fatty acid oxidation is converted into ketone bodies in the liver, leading to increased blood ketone levels. During starvation or the consumption of a low-carbohydrate diet, the balance between oxaloacetate and acetyl-CoA is disrupted as more oxaloacetate is consumed owing to the upregulation of gluconeogenesis, leading to a decline in energy metabolism. Therefore, if ketone bodies enhance muscle performance through the activation of the citric acid cycle, we hypothesize that this effect could be further amplified by oxaloacetate supplementation. To test this hypothesis, we investigated the physiological effects of oxaloacetate on myoblasts using a nutritional ketosis model and found that oxaloacetate synergistically enhanced the effects of ketone bodies in promoting myoblast differentiation.

During myoblast differentiation, the demand for energy increases, which stimulates mitochondrial biogenesis [10]. Notably, mitochondrial biogenesis declines with age and is implicated in the development of sarcopenia [22]. Our results suggest that oxaloacetate promotes myoblast differentiation by enhancing mitochondrial energy metabolism. This is evidenced by an increased mtDNA copy number, elevated expression of citrate synthase (*Cs*), and higher intracellular ATP content. Furthermore, the upregulation of *Ppargc1a* and *Tfam* gene expression further reflects enhanced mitochondrial biogenesis and function. It should be noted, however, that while an increase in Cs gene expression was observed, the enzymatic activity of citrate synthase was not assessed in this study. This represents a limitation, as the actual enhancement of mitochondrial oxidative capacity remains to be directly confirmed.

Although the changes in mtDNA copy number and intracellular ATP content had smaller effect sizes than those observed in gene expression, they may still be biologically important for elucidating the mechanisms underlying myoblast differentiation. In contrast, ketone bodies alone did not induce these mitochondrial changes; rather, they synergistically enhanced the effects induced by oxaloacetate. These findings suggest that ketone bodies promote myoblast differentiation by engaging an alternative, mitochondria-independent signaling pathway and by further augmenting the energy metabolism activated by oxaloacetate.

Activation of mTOR through the PI3K/Akt/mTOR signaling pathway promotes intracellular protein synthesis during myoblast differentiation [23]. mTOR activation is regulated by phosphorylation of Akt, an upstream effector of mTOR, leading to the formation of its active phosphorylated form (p-Akt). Elevated levels of p-Akt then stimulate mTOR activation, thereby promoting muscle cell differentiation [24]. Previous animal studies have reported that ketone bodies promote Akt phosphorylation in skeletal muscle [23]. Consistent with this finding, our study demonstrated that ketone bodies increase Akt phosphorylation in a cultured cell model of nutritional ketosis. Thus, it is suggested that ketone bodies promote myogenesis by promoting myoblast differentiation via Akt phosphorylation. On the other hand, our results suggest that oxaloacetate promotes myoblast differentiation without activating this pathway.

This study demonstrates that ketone bodies promote myogenesis through the activation of the PI3K/Akt/mTOR pathway, whereas oxaloacetate enhances myogenesis by stimulating mitochondrial biogenesis and the citric acid cycle to increase energy production. These findings suggest that ketone bodies and oxaloacetate influence myoblasts via distinct mechanisms and synergistically promote myogenic differentiation.

In this study, we consider that the synergistic effect of ketone bodies and oxaloacetate on myoblast differentiation is mediated by an increase in intracellular ATP content due to the activation of the citric acid cycle. This consideration is supported by the observed increase in Cs expression, mitochondrial DNA copy number, and expression levels of *Ppargc1a* and *Tfam*, markers of mitochondrial biogenesis. However, further investigations are necessary to more definitively confirm this mechanism. Given that the observed increase in intracellular ATP content may also stem from enhanced glycolytic activity, future studies should assess the oxygen consumption rate (OCR), quantify energy metabolism intermediates through metabolomic analysis, and evaluate not only the expression of citrate synthase (Cs) but also its enzymatic activity. These assessments would provide more direct functional evidence for the activation of the citric acid cycle.

Additionally, oxaloacetate synergistically enhanced the effects of ketone bodies on myoblast differentiation. However, the specificity of this effect to oxaloacetate remains unclear, considering its physiological relevance in humans. Oxaloacetate is a metabolic product that is formed when aspartic acid undergoes transamination and loses its amino group. As this reaction primarily occurs in the liver, we were unable to evaluate whether aspartic acid exerts effects similar to those of oxaloacetate using the single-tissue culture model in this study. When expanding this research to human intervention studies, the combination of oxaloacetate and ketone bodies as well as the potential effects of aspartic acid and ketone bodies on muscle formation should be assessed.

Furthermore, although myoblasts differentiate into myotubes in vitro, they do not mature into functional muscle fibers. Thus, the cell culture model employed in this study did not fully recapitulate the structural and functional aspects of the skeletal muscle. This limitation is not unique to this study but is a common constraint in in vitro myoblast differentiation models. Furthermore, since this study was conducted using an immortalized myoblast cell line, further validation with primary satellite cells—which more closely mimic in vivo myogenesis—is necessary to confirm the physiological relevance of our findings. In addition, considering the complexity of metabolic interactions among organs, future investigations employing in vivo models will be essential to more accurately assess the systemic effects of ketone bodies and oxaloacetate on muscle differentiation.

## 4. Materials and Methods

### 4.1. Cell Culture

L6 myoblasts (JCRB Cell Bank, Osaka, Japan) were plated at a density of 4 × 10^5^/ϕ 35-mm dish and a 6 × 10^5^/T12.5 flask with an oxygen-permeable cap (BD Falcon, Franklin Lakes, NJ, USA) and were then cultured in low-glucose DMEM (FUJIFILM Wako Pure Chemical Co., Osaka, Japan) with a 10% fetal bovine serum (Thermo Fisher Scientific, Waltham, MA, USA) for 4 d. Upon reaching a 70–90% subconfluence, the medium was replaced with a differentiation medium, which was either low- or high-glucose DMEM containing a 2% horse serum (Thermo Fisher Scientific) to induce myoblast fusion and differentiation into myotubes [25]. The cells were grown in a differentiation medium and were treated with or without ketone bodies (1 mM lithium-acetoacetate and 4 mM 3-hydroxybutyrate/hydroxybutyric acid), 16 mM oxaloacetate, citrate, α-ketoglutarate, and pyruvate. Myoblasts were cultured for 4 d in a differentiation medium, which was replaced every 2 d, for differentiation into myotubes. In this study, the control condition used to evaluate the effects of ketone bodies and oxaloacetate was cultured in a differentiation-inducing medium alone. All experimental treatments were compared to this standard differentiation condition to accurately assess their specific effects on myoblast differentiation and metabolic adaptation.

### 4.2. Cell Viability

The viability of cells cultured in the differentiation medium for 24 h was assessed using the neutral red assay, according to a previously established protocol [26]. A stock solution of 0.4% neutral red in water was diluted with phosphate-buffered saline (PBS) in a 1:80 ratio. The cells were then incubated with a neutral red solution for 30 min at 37 °C to allow viable cells to incorporate the dye into the lysosomes. To extract neutral red, the cells were treated with a solution of 1% acetate and 50% ethanol and incubated at room temperature for 30 min. The absorbance was measured at 540 nm using a microplate reader (Bio-Rad Laboratories, Hercules, CA, USA).

### 4.3. Real-Time Quantitative PCR

After washing the cells with PBS, total RNA was extracted using the Quick-Gene RNA Cultured Cell Kit S RC-S (KURABO, Tokyo, Japan). Equal quantities of RNA were reverse transcribed using a ReverTra Ace qPCR RT kit (TOYOBO, Osaka, Japan). Real-time quantitative PCR was performed on StepOnePlus (Thermo Fisher Scientific) using specific primers (Supplementary Table S1) and Thunderbird SYBR qPCR Mix (TOYOBO). Following were the PCR cycling conditions: 95 °C for 60 s, followed by 40 cycles of 95 °C for 15 s, and 60 °C for 35 s. mRNA levels were normalized to that of Actb, and data analysis was performed using the ΔΔCT method [27] with StepOne software v2.2.2 (Thermo Fisher Scientific).

### 4.4. Mitochondrial DNA Copy Number

Total DNA was extracted from L6 cells that had differentiated into myotubes after a 4-day culture in a differentiation medium using a NucleoSpin tissue kit (Macherey-Nagel, Düren, Germany) with proteinase K and RNase treatment. Specific primers (Supplementary Table S1) were used to amplify an mtDNA fragment encoded by *CO1* to determine the mtDNA content, and nuclear DNA encoded by *Actb* was used as an internal control.

### 4.5. Western Blot Analysis

The L6 cells were washed with PBS and lysed using a radioimmunoprecipitation as-say buffer supplemented with protease and phosphatase inhibitor cocktails (Nacalai Tesque, Kyoto, Japan). The lysates were placed on ice for 10 min and then centrifuged at 12,000× *g* for 10 min at 4 °C. Equal amounts of proteins were separated via sodium do-decyl sulfate-polyacrylamide gel electrophoresis and transferred onto a polyvinylidene fluoride membrane using a semi-dry blotting device. After blocking overnight with 1% (*w*/*v*) skim milk, the membranes were probed with anti-phospho-Akt, anti-Akt, anti-MHC IIb, Actb (Cell Signaling Technology, Danvers, MA, USA), and anti-Ckm (Proteintech Group, Inc., Rosemont, IL, USA) antibodies for 1 h at room temperature. After several washes with Tris-buffered saline with Tween 20 (TBS-T), the membranes were incubated for 1 h at room temperature with the appropriate horseradish peroxidase-conjugated secondary antibodies diluted in TBS-T with 1 or 2% (*w*/*v*) skim milk. Details regarding the antibody source, dilution ratio, and skim milk concentrations are provided in Supplementary Table S2. The bands were visualized using ImmunoStar LD (Fujifilm Wako Pure Chemical Co., Osaka, Japan) or EzWestLumi Plus (Atto Corporation, Tokyo, Japan).

### 4.6. ATP Content

L6 cells were cultured in 96-well plates at a concentration of 6.0 × 10^5^ cells/well. When cell growth reached a subconfluent level of 70% to 90%, treatments were applied in the presence or absence of ketone bodies (5 mM) and oxaloacetate (16 mM) over a period of 4 d. After the incubation period, ATP contents were determined using a CellTiter-Glo^®^ 2.0 assay (Promega, Madison, WI, USA) following the manufacturer’s guidelines.

### 4.7. Statistical Analysis

Data are presented as the mean ± SD from three or six independent experiments. Statistical analysis was conducted using one-way analysis of variance (ANOVA), followed by the Tukey–Kramer multiple comparison test. The synergistic effect of ketone bodies and oxaloacetate on myoblast differentiation was evaluated using two-way ANOVA. Analyses were performed using Statcel-4 software (OMS Inc., Saitama, Japan).

## 5. Conclusions

We demonstrated that ketone bodies enhance myoblast differentiation by establishing appropriate culture conditions in flasks equipped with hydrophobic membrane filters at physiological glucose concentrations. Additionally, we showed for the first time that oxaloacetate synergistically enhanced ketone body-induced myoblast differentiation. Ke-tone bodies and oxaloacetate enhance myogenesis, with the former enhancing Akt phosphorylation and the latter increasing energy production via mitochondrial biogenesis. Therefore, oxaloacetate may provide a new strategy for improving the efficacy of nutritional ketosis.

Sarcopenia is caused in part by impaired muscle differentiation due to malnutrition and aging. Hence, combined supplementation with ketone bodies and oxaloacetate may promote myoblast differentiation, potentially contributing to the prevention of sarcopenia.

## Data Availability

The original contributions presented in this study are included in the article/Supplementary Materials. Further inquiries can be directed to the corresponding author(s).

## Supplementary Materials

The following supporting information can be downloaded at: https://www.mdpi.com/article/10.3390/molecules30102101/s1, Table S1: Primer information used for real-time RT-PCR; Table S2: Antibody information used for western blot analysis.
