# Supplementary material for: Oxaloacetate and Ketone Bodies Synergistically Promote Myoblast Differentiation in L6 Cells
Source: Molecules. 2025 May 9;30(10):2101. doi: 10.3390/molecules30102101 (PMC12114476; doi:10.3390/molecules30102101)
Supplement: Supplementary file 1 [file molecules-30-02101-s001.zip › molecules-3528147-supplementary.pdf]

Table S1: Primer information used for real-time RT-PCR

Name	Sequence (5'-3') (F, forward; R, reverse)	Accession Number	Amplicon Length, bp
Lmod2	TAAGACAGCTACGGAGGGTGG<br>CAGCTCACAATGCACCTCAAG	NM_001100964.1	107
Ckm	GATTCTCACCCGCCTTCGT<br>AGCTTTACGCCATCCACCAC	NM_012530.2	147
Myod1	TCTCTCTGCTCCTTTGCGAC<br>CACCGTAGTGGGGAAGTGTG	NM_176079.2	153
Myog	GGTGGTACCCAGTGAATGCAA<br>AATGATCTCCTGGGTTGGGA	NM_017115.3	92
Cs	TAAGAACCCCTGGCCCAAC<br>TCGACACTCCGAACAGGACT	NM_130755.1	101
Tfam	AAGCTAAACACCCAGATGCAA<br>GCTCACAGCTTCTTTGTACACC	NM_031326.2	140
Ppargc1a	CAAGAGGGACGAATACCGCA<br>ACGGCGCTCTTCAATTGCTT	NM_031347.1	97
Actb	GGAGATTACTGCCCTGGCTCCTA<br>ACTCATCGTACTCCTGCTTGCTG	NM_031144.3	150
Mt Co1	ATCGCAATTCCTACAGGCGT<br>CTGTTAGGCCCCCTACTGTG	NC_001665.2	130
Actb	GGAGATTACTGCCCTGGCTCCTA<br>ACTCATCGTACTCCTGCTTGCTG	NM_031144.3	233

Table S2: Antibody information used for western blot analysis

Primary Antibody	Manufacturer	Primary Antibody Dilution	Secondary Antibody Dilution	Skim Milk Concentration (%)
Act β	Cell signaling	2500	2500	1
MHC Ⅱb	Cell signaling	1500	2000	1
Ckm	Proteintec	1000	2000	2
Cs	Cell signaling	1500	4000	1
Akt	Cell signaling	1500	2000	1
p-Akt	Cell signaling	1000	2000	1
